# Nurses in advanced roles as a strategy for equitable access to healthcare in the WHO Western Pacific region: a mixed methods study

**DOI:** 10.1186/s12960-021-00555-6

**Published:** 2021-02-15

**Authors:** Sue Kim, Tae Wha Lee, Gwang Suk Kim, Eunhee Cho, Yeonsoo Jang, Mona Choi, Seoyoung Baek, David Lindsay, Sally Chan, Regina L. T. Lee, Aimin Guo, Frances Kam Yuet Wong, Doris Yu, Sek Ying Chair, Yoko Shimpuku, Sonoe Mashino, Gigi Lim, Sheila Bonito, Michele Rumsey, Amanda Neill, Indrajit Hazarika

**Affiliations:** 1 College of Nursing, Mo-Im Kim Nursing Research Institute, Yonsei University, 50-1 Yonsei-ro, Seodaemun-gu, Seoul, 03722 Republic of Korea; 2 Korea Armed Forces Nursing Academy, Jaun-ro 90, Yuseong-gu, Daejeon, 34059 Republic of Korea; 3 James Cook University, 1 James Cook Drive, Douglas, Townsville, QLD 4811 Australia; 4 Tung Wah College, 31 Wylie Road, Kowloon, Hong Kong SAR China; 5 The University of Newcastle, University Drive, Callaghan, NSW 2308 Australia; 6 School of Nursing, Peking Union Medical College, No.9 Dong Dan San Tiao, Dongcheng, District, Beijing, 100730 China; 7 The Hong Kong Polytechnic University, Hung Hom, Kowloon, Hong Kong SAR China; 8 The School of Nursing, LKS Faculty of Medicine, The University of Hong Kong, 21, Sassoon Road, Pokfulam, Hong Kong SAR China; 9 The Chinese University of Hong Kong, Shatin, NT, Hong Kong SAR China; 10 Hiroshima University, 1-2-3 Kasumi, Minami-ku, Hiroshima City, Hiroshima 734-8551 Japan; 11 Research Institute of Nursing Care for People and Community, University of Hyogo, 13-71 Kitaohji-cho, Akashi, Hyogo 673-8588 Japan; 12 The University of Auckland, Private Bag 92019, Auckland, 1142 New Zealand; 13 College of Nursing, University of the Philippines Manila, Pedro Gil St, Ermita, 1000 Manila, Metro Manila, Philippines; 14 University of Technology Sydney, 15 Broadway, Ultimo, NSW 2007 Australia; 15 WHO Ethiopia Country Office, Menelik Avenue, PO Box 3069, Addis-Ababa, Ethiopia

**Keywords:** Advanced practice nursing, Health equity, Health services accessibility, Nurse²s role, Professional role

## Abstract

**Background:**

The Western Pacific region constitutes one-quarter of the world’s population and has diverse health needs. While dialogue on and promotion of advanced practice nurses are ongoing, this study investigated the current responsibilities of nurses in advanced roles, future healthcare needs, and the implications of these components for nurses’ professional development within the Western Pacific region.

**Methods:**

This study employed three phases, a descriptive survey on the current status of nurses in advanced roles in the Western Pacific region, followed by a Delphi survey, and exploratory interviews. A total of 55 national experts with clinical, academic, and/or government-related backgrounds from 18 countries participated from December 2017 – December 2018. The descriptive survey via email to identify the status of nurses in advanced roles and a working definition was developed. This formed the basis for the Delphi survey, which identified key barriers and challenges for enhancing the development of nurses in advanced roles within the country (round 1) and for the region (rounds 2 and 3). Lastly, semi-structured individual interviews were conducted to identify strategies for establishing nurses in advanced roles to improve equitable access to healthcare.

**Results:**

Thirty-seven roles and characteristics were identified and categorized for nurses performing advanced roles. Emergency care, critical care, elderly health, child health, and rural/remote communities were identified as fields with particular need for nurses in advanced roles in the Western Pacific region. Providing effective services, influencing government leadership, and advocating for health system sustainability were deemed necessary to improve equitable healthcare access. We found that nurses in advanced roles are not limited to clinical tasks within the hospital but are poised for active participation in primary healthcare, education/teaching, professional leadership, quality management, and research.

**Conclusions:**

Demand for nurses in advanced roles is high in the Western Pacific region and 15 items were identified across five core strategic areas to enhance development of nurses in advanced roles. Governmental-level recommendations include establishing legislative protection, improving systems for remuneration, strengthening supportive channels, and conducting national needs assessments.

## Introduction

The Western Pacific region (WPR) of the World Health Organization (WHO) includes almost 1.9 billion people in 37 countries and areas, constituting one-quarter of the world’s population [1]. The changing demographic and epidemiological trends in WPR countries have resulted in a shift in population healthcare needs, creating an increased demand for health services. Regardless of socio-economic development levels, there is a growing global recognition of the importance of the health worker’s role in promoting equitable access to quality health services. In most countries, nurses represent by far the largest part of the healthcare workforce and are critical to universal health coverage (UHC) [2] and the Sustainable Development Goal (SDG) agendas [3].

Within the nursing workforce, advanced nursing roles such as nurse practitioners (NP), advanced practice nurses (APN), and midwives have evolved to provide high quality care, especially at the primary care level. The International Council of Nurses (ICN) has viewed NP/APN as interchangeable terms as seen in the 2008 definition of “NP/APN is a registered nurse who has acquired the expert knowledge base, complex decision-making skills, and clinical competencies for expanded practice, the characteristics of which are shaped by the context and/or country in which s/he is credentialed to practice”, and specified that a master's degree is recommended for entry level [4]. These titles and subsequent roles have been embraced and developed in Northern America and other high-income countries such as the UK, the Netherlands, Finland, etc., with legislative protection and graduate-level education taking root [5, 6]. ICN’s recently updated guidance on APN currently specifies APN as “a generalist or specialised nurse who has acquired … the expert knowledge base, complex decision-making skills and clinical competencies for advanced nursing practice’ while clarifying a full masters’ degree as minimum education [7] The International Confederation of Midwives (ICM) defines a midwife as a person who has successfully completed a midwifery education programme that is based on the ICM Essential Competencies for Basic Midwifery Practice and the framework of the ICM Global Standards for Midwifery Education [8].

There is a sizeable body of empirical literature supporting the unique contributions of NP/APN and midwives from various regions around the world, including North America [9, 10], Latin America [11], Australia [12], and the Eastern Mediterranean region [13]. While these titles are widely known and specific to country context, they have not been sufficient nor perhaps relevant to countries with different health systems, resources, and historical contexts. Despite the fact that there are numerous nurses who take on a variety of roles and responsibilities extending beyond their basic training and contributing considerably to healthcare throughout the world, nurses in advanced roles (NAR) have lacked consistency in nomenclature, scope of practice, role-related regulation, qualification, and educational systems. There is limited empirical literature aside from the aforementioned countries where NP/APN are well established. Furthermore, there has been a lack of concerted effort to examine the status and spectrum of NAR and identify potential contributions, especially in relation to the five essential health system attributes required to achieve UHC, i.e. quality, efficiency, equity, accountability, and sustainability and resilience [2].

## Methods

### Specific aims

Thus, the purpose of this study was to describe what NAR means in the context of the WPR countries, their current roles and contributions to health services in each country, as well as implications for the professional development of NAR to meet future healthcare needs at the national and regional levels. The specific objectives were to (1) identify the current status of NAR in the WPR (e.g. functions, scope, competencies, educational standards, credentialing, and regulation); (2) assess how NAR might be able to improve equitable access to quality healthcare, including the identification of key barriers and challenges; and (3) identify the role of NAR in addressing future healthcare needs, including recommendations on their contributions and roles in the healthcare system. This manuscript followed the STROBE reporting guidelines for observational research and COREQ for qualitative research.

This multi-country study was conducted by the NAR Study Group (13 institutions from 8 countries), formed from a previously existing network of nursing and midwifery related WHO Collaborating Centers (CCs), from December 2017 to December 2018, in three phases: A descriptive survey, a Delphi survey, and exploratory interviews, followed by analysis and integration of data. Coordination of the study was done by the Working Group at the College of Nursing, Y University, Seoul, Korea. Following ethical approval from the Institutional Review Board of Y University Health Systems (Y-2017-0076), an information sheet and consent form were sent to participants at each phase via email and signed consent forms were obtained.

Participants for each phase were recruited using purposive and snowball sampling to invite experts and key stakeholders who were identified by the NAR Study Group (Table 1). These included healthcare professionals (nurse clinicians), leaders (e.g. policy makers, experts in government positions), regional experts, and members of related networks, such as the South Pacific Chief Nursing and Midwifery Officers Alliance (http://www.spcnmoa.com/). They were recommended by Study Group members as experts knowledgeable about nurses’ roles, especially regarding various advanced roles, and were invited to the study by the Working Group.

**Table 1:** Participants in each phase

Participants	Descriptive survey (Phase 1)	Delphi method (Phase 2)	Exploratory interview (Phase 3)
	*n* = 15	*n* = 27	*n* = 19
Australia	2	3	2
Cambodia*	0	1	0
China	3	3	2
Cook Islands*	0	0	1
Fiji*	1	0	1
Hong Kong	2	2	2
Japan	1	2	1
Kiribati*	0	0	1
Korea	1	3	2
Laos PDR*	0	2	0
Malaysia*	0	3	0
New Zealand	1	1	3
Philippines	2	3	2
Singapore*	0	1	0
Solomon Islands	1	1	0
Tonga*	0	0	1
Viet Nam*	0	1	0
Vanuatu*	1	1	1
18 countries	10 countries	14 countries	12 countries

* Countries other than the study team

### Phase 1: descriptive survey

This first step aimed to gather descriptive data on the spectrum of NAR for each country, to form a working definition of NAR that could guide the overall study. In the current study, as we wanted to go beyond traditional conceptualization attached to NP/APN and capture the spectrum of roles and responsibilities according to WPR country contexts, the term “NAR” was intentionally used very broadly to describe nurses undertaking roles that are beyond the basic level of nursing. The NAR Study Group identified 20 experts (policy makers, academics, leaders of nursing organizations) who were knowledgeable about the state of NAR pertaining to their country, and 15 experts (response rate 75%) from 10 countries participated from December 2017—March 2018. The email survey, constructed from the literature, consisted of two parts: (1) the status of NAR in their countries, e.g. role/title names (in English as well as the phonetic expression in the original language), role-related regulations, credentialing, and education preparation. These items were open-ended questions so that respondents could answer according to what NAR meant for each country; (2) NAR’ specific roles and responsibilities in 6 areas (29 items) derived from the literature: clinical/technical tasks (11 items), primary care (8 items), education and teaching (4 items), professional leadership (2 items), quality management (2 items), and research (2 items). Reminders were sent up to three times via email to ensure responses. Based on this expert input, a working definition of NAR was developed to guide the subsequent phases of the study.

### Phase 2: Delphi method

The Delphi method [14] was employed to identify consensus among experts on how NAR roles might be able to improve equitable access to quality healthcare, including identification of key barriers/challenges and roles for NAR in achieving UHC. Experts who had not participated in Phase 1 were invited to gain better understanding of the diverse mix of countries within WPR, additional countries were also included. A total of 27 experts from 14 countries participated from April–July 2018. The working definition of NAR was presented to respondents, and experts were initially asked to rate the status of NAR within their country. Rounds 2 and 3 were expanded to focus on rating how NAR could contribute to improving equitable access to quality healthcare within the WPR.

The Delphi questionnaire was developed from the literature by the Study Group and consisted of three parts: (1) Areas in need of NAR (26 items), which was divided into four sub-domains (field-specific domain, client group-specific domain, area-specific domain, and professional role-specific domain), rated on a five-point Likert scale (1 = *not required*, to 5 = *essential function for NAR*). (2) Positive and negative influences in developing and establishing advanced nursing roles were assessed using the SWOT (Strengths, Weakness, Opportunities, and Threats) format [15]. Thirty-eight items were rated on a five-point Likert scale (1 = *strongly disagree*, to 5 = *strongly agree*) as well as “not applicable”. (3) Lastly, priority of the role of NAR in UHC development (15 items) was assessed according to the five WHO domains (2016)—quality, efficiency, equity, accountability, and sustainability/resilience—on a five-point Likert scale (1 = *low priority*, to 5 = *high priority*).

For round 3 the mean and range of round 2 responses were presented to the experts as reference data, and they were asked to answer considering this information or write down the reasons if their chosen response would be out of the range.

### Phase 3: exploratory interviews

**Fig. 1:**
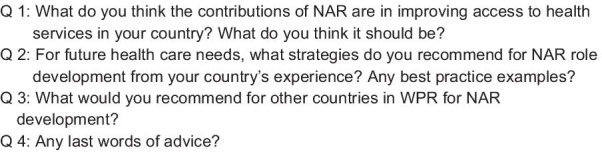
Interview guide used for Phase 3

This phase sought to explore and understand how NAR improve access to health services with country examples and to solicit expert recommendations for NAR across various health systems to improve equitable access to healthcare in the WPR. Key informants were identified by the Study Group members, e.g. nurses in leadership positions (hospital and/or community-level), academia, and regional experts. Semi-structured individual interviews with 19 key informants from 12 countries were conducted from July–September 2018. With the exception of a few who had also participated in Phase 1, the majority were newly identified experts. Interview questions are presented in Fig. 1.

Twelve Study Group members conducted face-to-face or telephone interviews in the language of the informant to obtain relevant and accurate data, with interviews ranging from 30 to 90 min. While each interview was transcribed by the interviewer in their language, due to the diverse spectrum of languages, an English summary for each interview was shared among the Group for discussion. In the process of content analysis we discussed and clarified intended meanings/nuances through email and online discussions.

### Phase 4: analyses and integration of phases 2 and 3

Descriptive statistics were done for the descriptive survey (Phase 1) and Delphi (Phase 2). For the Delphi phase, to identify differences in areas in need of NAR according to national income level, each country’s income category [16] was re-grouped for comparison purposes, with low-income and lower-middle-income economies grouped together as low-income countries, and upper-middle-income and high-income economies categorized as high-income countries. Thus, for this study high-income countries included Australia, China, Hong Kong, Japan, and South Korea; low-income countries included the Philippines, Vanuatu, the Solomon Islands, etc.

For the qualitative data from Phase 3, content analysis was performed according to the existence of NP-related regulation by country as well as by inductively identifying domain areas. The two focus areas of content analysis were: 1) areas in which NAR were currently contributing to improve access to healthcare in each country and how they can contribute in the future, and 2) strategies for increasing the use of NAR in each country as well as within the WPR at large.

The six-member NAR Working Group of Y University drafted plans for analysis for each phase, which were verified by the Study Group. Following general agreement, the Working Group analysed the data over 14 research meetings and interim results were reviewed by and revised with input from the larger Study Group through multiple teleconferences and email exchange. Further verification of the data and findings were also shared in face-to-face meetings with some research partners during international conferences and visits. Finally, integrated results of the Delphi and exploratory interview phases were reviewed by the Study Group to derive strategies and recommendations for developing and promoting the role of NAR in the WPR.

## Findings

### Phase 1: status of health systems and NAR in the WPR

We identified 37 role domains and characteristics of NAR, which were not limited to clinical tasks within the hospital but also included primary healthcare, education/teaching, professional leadership, quality management, and research.

While 8 countries had role-related regulations, only 4 countries had roles that were consistent with the definitions put forth by the ICN NP/APN network [4]: NPs in Australia, New Zealand, and Fiji; and specialist nurses or public health officers in the Solomon Islands. There were also legally stipulated roles although these did not necessarily meet the definition put forth by the ICN. These were APN (midwife specialist) role or midwife consultants in Hong Kong; public health nurses or midwives in Japan; specialized nurses (home healthcare) or public officials exclusively responsible for public healthcare services in South Korea; clinical nurse specialists with prescribing rights in New Zealand; and in the Philippines, clinical nurse specialists based in hospitals (e.g. enterostomy nurses, geriatric nurses) or potential NPs in communities (e.g. nurse-midwives, nurse diabetes educators). For NAR lacking regulatory standards in countries such as China and Vanuatu, they were described in standards by nursing and professional associations, job descriptions, and/or training standards by educational institutions. The minimum level of education required differed depending on the role and country, ranging from an advanced diploma to a master’s degree.

Based on the data, the following working definition of NAR was formulated for use in the current study:

### Phase 2: areas in need of NAR and key barriers and challenges to developing the functions of NAR

#### Delphi Round 1 on country-level needs

Emergency care (4.82 ± 0.53), and critical care and mental healthcare (both 4.76 ± 0.44) were identified as top essential areas for NAR.

While emergency care was a common high priority, comparison by economic levels showed slight differences. For high-income countries there was a high demand for mental health and elderly health; whereas critical care, communicable disease control, and maternal health/midwifery were in high need in low-income countries. Clinical leadership, professional leadership, and research roles were also noted as high priority areas in the latter.

SWOT results showed that the greatest strengths contributing to NAR development were accessible education and training systems (4.06 ± 1.03) and the effort of national/state nurses associations (4.06 ± 1.14). In contrast, the greatest weaknesses were absence or ambiguity of a systematic career path for NAR (4.00 ± 1.12) and lack of funding sources within nursing to pursue advanced role education (3.94 ± 1.09). While respondents viewed changing demand for care (4.59 ± 0.71) and general definition of the scope of practice of NAR in legislation (4.41 ± 0.87) as possible opportunities, they noted the lack of regulations specifying roles (4.41 ± 0.94), and lack of positions available/created for NAR (4.41 ± 0.71) as threats.

Regarding NAR role contributions to UHC, highest scoring items were ‘providing effective, responsive individual and population-based services ensuring accessibility and availability’ (4.88 ± 0.33; Quality domain), ‘participating in partnerships for public policy’ (4.76 ± 0.56; Accountability domain) and ‘advising policy on incentives for appropriate provision and use of services’ (4.65 ± 0.61; Quality domain).

#### Delphi Rounds 2 and 3 on regional-level needs

While emergency care continued to remain a high priority area, elderly health and rural/remote areas emerged as essential areas for NAR (3.95 ± 1.08 to 4.00 ± 1.05) for the WPR compared to country-level responses.

Regarding the positive and negative influences in developing NAR roles, ‘accessible education and training system’ (4.74 ± 0.56) was the highest scoring strength, and ‘weak or absent leadership and advocacy by professional nursing organization’ (4.42 ± 0.84) was the highest scoring weakness. ‘Changing demand for care’ (4.58 ± 0.83) was the highest scoring opportunity factor, and ‘lack of regulation specifying roles’ (4.58 ± 0.77) was the highest scoring threat. Considering both the positive and negative aspects of the internal and external environments identified through SWOT analysis, strategies for developing NAR roles were prioritized.

For NAR towards UHC within WPR, consensus was that the accountability domain demanded highest priority, followed by the sustainability and resilience domain and the quality domain.

### Phase 3: policy recommendations to increase the use of NAR to improve access to health services in the WPR—interview findings

Experts noted that NAR know the community and are respected role-models that provide various high quality healthcare services, e.g. operating independent clinics and providing patient-centred care, while often being the quickest contact-point for clients, contributing to overall healthcare improvement.

For strategies for NAR role development, experts from countries with NP/APN role-related regulation in place emphasized that “NAR roles must be recognized by other professionals and/or organizations” and “NAR need to develop specific abilities, such as policy-making, communication, and negotiation skills”. Conversely, in countries lacking role-related regulations, the need to increase positions was emphasized, such expanding nurses’ role from the hospital to the community, creation of positions in the government and private sector, and the importance of needs assessments to identify the clinical areas and communities where NAR are most needed.

Finally, at the WPR level, a three-level strategic framework to enhance the development of NAR roles was identified—micro-level (individual nurse/nursing group), organizational level, and macro-level (governmental). Micro-level strategies included increased opportunities for education, training, leadership/management capacity building, and conducting research. Organizational level strategies involved establishing clear paths of a career ladder system and developing stronger networking systems at the regional level. Recognizing the diversity within the WPR, specific examples included creating a regional association, networking to promote research on NAR, and strengthening collaboration between countries to share experiences and learn from each other. Macro-level strategies included increasing remuneration for higher-level roles, legislation and policy support for NAR, vision and support from organizations/governments, and conducting assessments to determine where NAR are most needed.

### Phase 4: integration of phases 2 and 3

**Fig. 2:**
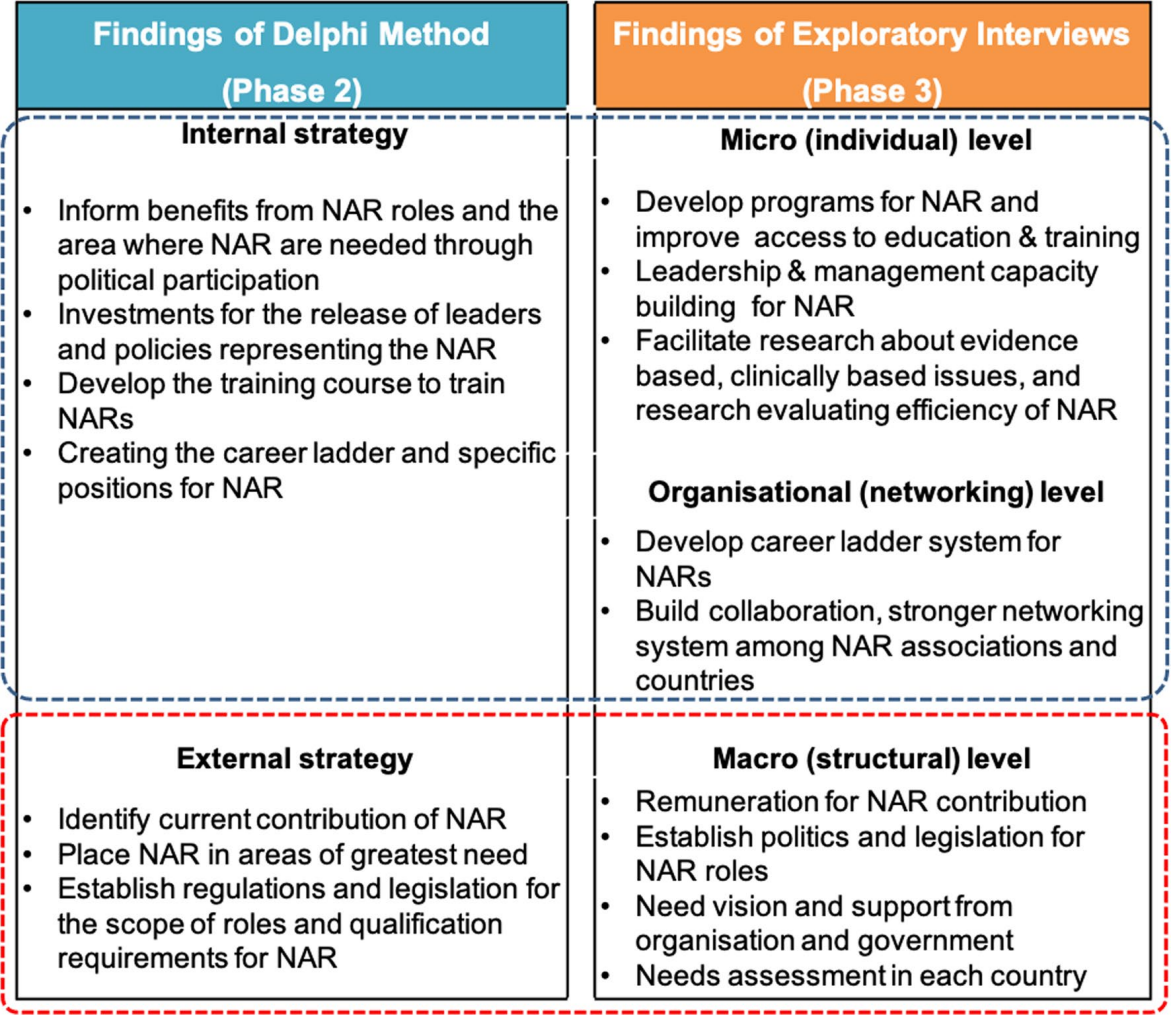
Strategies to enhance the development of NAR identified from phases 2 and 3

**Fig. 3:**
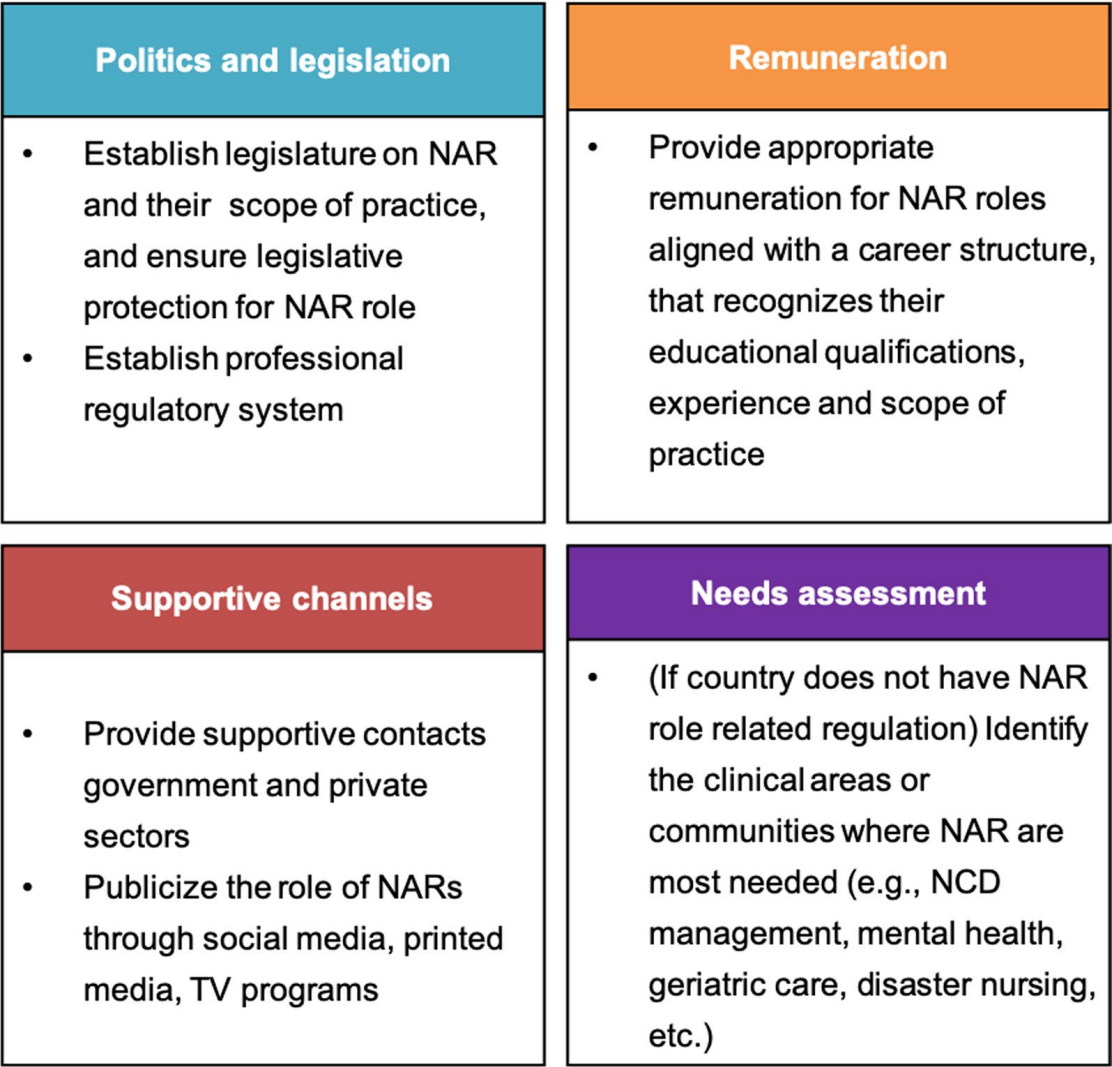
Governmental-level recommendations for the promotion of NAR

Upon integration, strategies derived from the Delphi phase aligned with the content analysis results of the exploratory interviews (Fig. 2). The Delphi internal strategies were found to be parallel to the micro- and organizational levels of the exploratory interviews, while external strategies of the Delphi phase were similar in context to macro-level findings of the interviews. Thus, five core strategic recommendation areas were extracted within the nursing domain (Fig. 2) and governmental-level recommendations for promoting NAR in WPR (Fig. 3) were identified.

## Discussion

This study has some limitations, one being that not all 37 WPR countries were included in this study and a greater number of experts (2–3 per country) participated from the Study Group countries as opposed to the other countries (1–2 per country). As such, findings should be interpreted with caution to other WPR countries. There may also be potential variation in interpreting the advanced role types that emerged from Phase 1 data, as experts participating in Phases 2 and 3 sometimes appeared to answer referring to different types of NAR even if they were from the same country. It is possible that the scope of responses may have varied because the perception of the meaning of NAR differed, or the experts did not recognize working areas outside of their own field and/or setting. As English summaries of the exploratory interviews were shared there was a limit to the richness of narratives that could be attained, possibly affecting analyses of all pertinent information.

Despite these limitations, this study covers roughly half of the WPR (18 countries), involving national experts from various fields, e.g. healthcare professionals, policy makers, nurse leaders, and educators, and is significant as it is the first multi-national study focusing on how NAR can contribute to equitable healthcare access in the WPR. Also, systematic discussions with study partners were followed upon each stage to ensure accuracy.

This study shows that NAR vary in nomenclature, functions, legislation, education level, and qualifications by each country. Similar reports have been made in prior studies on advanced nursing practice, although they mostly lacked inclusion of WPR countries, especially those with lower income levels [17, 18]. In particular, there were a number of countries that had no specific regulations at the national level but followed the standards set by a national nursing association or job descriptions by educational institutions or employing bodies, which is similar to the findings of a previous study that targeted advanced nurse practitioners [19]. For WPR countries and areas that face diverse challenges (e.g. many island countries dealing with natural disasters with limited health resources), delineating NAR practice areas and regulations for nurses with appropriate expertise may be more relevant than such differentiation efforts. Considering new terminology (i.e. NAR) can highlight the important work of nurses and purposefully seeking ‘minority voices’ in the continuing dialogue on NP/APN may also expand prior conceptions, thus empowering nursing globally.

In several previous studies, legislation, educational development, and credentialing have been emphasized as crucial for the development of NP/APN [19, 20]. Strategies from our study are parallel to various strategies proposed for developing specialized NP roles in Canada [21]. Given that collaboration among nurse performing advanced roles and responsibilities improves role integration, autonomy, role clarity, and team capacity [9, 22], the emphasis on collaboration among organizations and countries noted in this study is a significant point. Although we propose region-wide strategies, considering differences in context, such as health systems, health status, income level, and status of NAR, further country-specific strategies can be identified and adapted for practical use through a platform for systematic sharing. An example may be establishing a regional taskforce linking national nursing networks working on NAR, supported by the General Network of WHO CCs related to Nursing and Midwifery, with close collaboration with global nursing professional organizations such as ICN and ICM.

Study participants emphasized that NAR are well positioned to meaningfully contribute to the achievement of UHC, an important SDG, especially in the domains of accountability, sustainability/resilience, and quality. As UHC is a high priority area of WHO and its WPR office [23], NAR can be further developed as a specific group within the healthcare workforce.

In essence, we were able to draw five core areas and 14 strategies within the nursing domain to assist in the development of NAR roles both at the organizational level (i.e. department of nursing within a hospital) and relating to professional nursing associations (Additional file 1: Table S1). In conjunction with improving nursing education/training, conducting research on NAR, creating career development pathways, enlisting multidisciplinary support, and building cross-country collaboration, government-level involvement and degree of commitment will ultimately promote or hinder NAR potential contributions to the health. If governments recognize the potential of this sizable health professional group, action is required to take a foundational step is establishing legislation on NAR and their scope of practice, and creating a professional regulatory system. Second, appropriate remuneration measures for NAR that align with career structures will strengthen the infrastructure for NAR development and sustainability. Third, establishing supportive channels within government and private sectors and improving public understanding of NAR through media and social network platforms has the power to increase NAR involvement in the community and at the policy table. Finally, as rapid shifts in health issues and demographics are increasingly being observed, comprehensive national needs assessments are required to carefully examine priority areas for NAR, which can then guide educational preparation at the national level [24].

## Conclusions

We found that demand for NAR is high in the WPR and presented recommendations for both the nursing community and at governmental-level to enhance NAR development and contributions for equitable healthcare access. We suggest further research that collates and highlights best practices for how NAR development and collaboration are best realized within diverse sociocultural contexts, especially in relation to UHC at local and regional levels. Also research that coordinates dialogue among nurses in clinical and/or policy positions, academia, and international organizations, is needed to clarify advanced nursing roles across NP, APN, and NAR.

## Supplementary Information

**Additional file 1: Table S1. **Strategic recommendations for the development of NAR within the nursing domain.

## Data Availability

The data generated and analysed for this study are available from the corresponding author on reasonable request.

## Abbreviations

APN: Advanced practice nurse
ICN: International Council of Nurses
ICM: International Confederation of Midwives
NAR: Nurses in advanced roles
NP: Nurse practitioner
PHC: Primary health care
SDG: Sustainable Development Goals
SWOT: Strengths, weaknesses, opportunities, threats
UHC: Universal health coverage
WHO: World Health Organization
WPR: Western Pacific region
