# Supplementary material for: Nurses in advanced roles as a strategy for equitable access to healthcare in the WHO Western Pacific region: a mixed methods study
Source: Hum Resour Health. 2021 Feb 15;19:19. doi: 10.1186/s12960-021-00555-6 (PMC7885366; doi:10.1186/s12960-021-00555-6)
Supplement: Supplementary file 1 — Additional file 1: Table S1. Strategic recommendations for the development of NAR within the nursing domain. [file 12960_2021_555_MOESM1_ESM.docx]

Supplemental table. Strategic recommendations for the development of NAR within the nursing domain

| **Domain** | **Strategic recommendations** |
| --- | --- |
| **Education**  **& Training** | 1. Master’s degree (or a doctorate) is recommended 2. Create a structured program through a nursing school or training institutions 3. Train for specific abilities (e.g., decision-making, leadership, budgeting, negotiation skills, etc.)  * This is also expected to contribute to achieve UHC 4. Offer training in other countries or visiting trainers from developed countries if resources are insufficient |
| **Research** | 1. Facilitate research about evidence-based, clinically-based issues 2. Conduct research about NAR to evaluate efficacy and highlight improvements in clinical outcomes. |
| **Career**  **Development** | 1. Clarify career progression (e.g., career ladder system) of nurses at organizational level 2. Create pathways for nurses to go into various positions (e.g., health administration, primary healthcare, government, private sector, academia, etc.)^a^ |
| **Multidisciplinary**  **Approach** | 1. Strengthen multidisciplinary teams (including nurses, physicians, and other health providers) or cross-cluster meetings from planning to decision-making 2. Build recognition of NAR from other professionals and organizations; Identify supportive sources from organizations and government ^a^ |
| **Collaboration**  **across Countries** | 1. Identify and link with support for NAR at regional level 2. Develop collaborative efforts between countries to share experiences and learn from each other (e.g., developing programs and practice) 3. Create an association for NAR regionally as well as globally |

^a^ If the country does not have NAR role related regulation.
